# Lyssavirus P-protein selectively targets STAT3-STAT1 heterodimers to modulate cytokine signalling

**DOI:** 10.1371/journal.ppat.1008767

**Published:** 2020-09-09

**Authors:** Angela R. Harrison, Kim G. Lieu, Florence Larrous, Naoto Ito, Hervé Bourhy, Gregory W. Moseley

**Affiliations:** 1 Department of Microbiology, Monash Biomedicine Discovery Institute, Monash University, Clayton, Victoria, Australia; 2 Department of Biochemistry and Molecular Biology, Bio21 Institute, University of Melbourne, Parkville, Victoria, Australia; 3 Lyssavirus Epidemiology and Neuropathology Unit, Institut Pasteur, Paris, France; 4 Laboratory of Zoonotic Diseases, Joint Department of Veterinary Medicine, Faculty of Applied Biological Sciences, Gifu University, Gifu, Japan; Washington University in Saint Louis School of Medicine, UNITED STATES

## Abstract

Many viruses target *signal transducer and activator of transcription* (STAT) 1 to antagonise antiviral interferon signalling, but targeting of STAT3, a pleiotropic molecule that mediates signalling by diverse cytokines, is poorly understood. Here, using lyssavirus infection, quantitative live cell imaging, innate immune signalling and protein interaction assays, and complementation/depletion of STAT expression, we show that STAT3 antagonism is conserved among P-proteins of diverse pathogenic lyssaviruses and correlates with pathogenesis. Importantly, P-protein targeting of STAT3 involves a highly selective mechanism whereby P-protein antagonises cytokine-activated STAT3-STAT1 heterodimers, but not STAT3 homodimers. RT-qPCR and reporter gene assays indicate that this results in specific modulation of interleukin-6-dependent pathways, effecting differential antagonism of target genes. These data provide novel insights into mechanisms by which viruses can modulate cellular function to support infection through discriminatory targeting of immune signalling complexes. The findings also highlight the potential application of selective interferon-antagonists as tools to delineate signalling by particular STAT complexes, significant not only to pathogen-host interactions but also cell physiology, development and cancer.

## Author summary

Lyssaviruses cause rabies, an almost invariably lethal progressive encephalomyelitis responsible for > 60,000 human deaths annually, with no antiviral treatments available. Evasion of host immunity is critical to infection/disease and involves potent suppression of signalling by interferons. However, suppression of responses to other important immune mediators by lyssaviruses and other human pathogenic viruses is poorly understood. Here, we show that the immune antagonist P-protein of diverse pathogenic lyssaviruses inhibits responses activated by interleukin-6 family cytokines, critical players in inflammatory responses, *via* targeting of the host transcription factor STAT3. Importantly, we find that this involves selective targeting of particular STAT3 signalling complexes, such that P-protein differentially modulates expression of certain interleukin-6-activated host genes. These findings provide new insights into how viruses can ‘fine-tune’ host immune responses, and into the biological functions of STAT3-complexes, which are currently poorly defined but are significant to immunity, cell physiology and pathologies such as cancer.

## Introduction

Rabies is an incurable neurological disease that disproportionately affects children and kills over 60,000 people every year [1–3]. The disease is caused by members of the *Lyssavirus* genus of highly pathogenic non-segmented single-stranded negative-sense RNA viruses, which have been detected on every continent except Antarctica, and include the broadly distributed rabies virus (RABV), as well as Mokola (MOKV) and Duvenhage (DUVV) viruses (identified in Africa), and Australian bat lyssavirus (ABLV) [4]. Lyssaviruses can be transmitted to humans by bites or scratches from infected animals and move through peripheral neurons to infect the central nervous system. Upon reaching the brain, the virus undergoes rapid replication, causing acute and progressive encephalomyelitis [1, 3]. In humans, symptomatic rabies is almost invariably fatal, with no antiviral treatments available or in clinical development. Vaccines in general use for humans and dogs use inactivated virus, and can be effectively applied pre- or post-exposure in humans, provided they are delivered before symptoms develop [3]. However, issues including high cost, a regimen of multiple injections for humans, and the need for delivery of rabies immunoglobulin in post-exposure prophylaxis limit use, particularly in developing nations where most human rabies occurs [1, 2]. Thus, there exists a need to investigate the mechanisms underlying rabies to identify new targets for antiviral drug development or the design of new live-attenuated virus vaccines, which could contribute to control in dogs, the major source of human infection.

In common with other mammalian viruses, evasion of the host antiviral interferon (IFN)-mediated immune response is critical to RABV infection [5–8] and is mediated by virus-encoded IFN-antagonist proteins. A major function of many IFN-antagonists, including the principal RABV antagonist phosphoprotein (P-protein), is the targeting of *signal transducers and activators of transcription (STATs)* 1 and/or 2, the major mediators of signalling by antiviral type I IFN (IFN-α/β) that represents the principal innate antiviral response [9, 10]. The STATs comprise a family of 7 proteins (STAT1-4, STAT5a and STAT5b, and STAT6) that are activated in response to cytokines and other stimuli by phosphorylation at a conserved C-terminal tyrosine. This leads to the formation of parallel dimers, which accumulate within the nucleus and bind to specific DNA elements in the promotor regions of large numbers of target genes, producing distinct profiles depending on the cytokine stimulus and specific STATs activated [11]. RABV P-protein binds directly to STAT1 *via* the P-protein C-terminal domain (CTD) [6, 8, 12], with efficient interaction in cells requiring activation by cytokines [13]. Thus, P-protein interacts strongly with tyrosine-phosphorylated (pY) STAT1-STAT2 heterodimers in IFN-α/β-treated cells, and inhibits nuclear accumulation *via* a nuclear export sequence in the P-protein N-terminus (N-NES), which effects cytoplasmic localisation of the complex [5, 12–14]. P-protein similarly binds and inhibits STAT1 homodimers, the major complex in type II IFN (IFN-γ)-activated cells [12, 14]. STAT1 interaction and N-NES activity is conserved across the *Lyssavirus* genus [15] and highly significant to disease progression *in vivo* [5, 6, 8].

Viral subversion of STAT1/2 signalling is well appreciated due to major roles in the IFN-mediated antiviral response, but an increasing number of viral antagonists are reported to bind/inhibit other STATs including STAT3. STAT3 antagonists include V-protein of several paramyxoviruses [16–18], IE1 of human cytomegalovirus [19], NSP5 of porcine reproductive and respiratory syndrome virus [20], cyclin K of Kaposi’s sarcoma-related herpesvirus [21], and P-protein of RABV [22]. STAT3 is activated by IFN-α/β, contributing to the IFN signalling profile, but is also the major mediator of Gp-130-receptor signalling by interleukin-6 (IL-6) cytokine family members [23]. Thus, antagonism of STAT3 is expected to impact an array of innate immune cytokines. Of note, several viral IFN-antagonists that target STAT1 do not target STAT3, and *vice versa*, indicating that interactions are highly specific and virus-dependent [16, 18, 24]. The extent of direct targeting of STAT3 among viruses, however, is poorly characterised, and conservation in lyssaviruses other than RABV is not known.

The diverse cellular roles of STAT3 mean that it is likely to be able to exert antiviral or ‘proviral’ effects for particular viruses (reviewed in [25, 26]), and many oncoviruses activate STAT3 signalling to influence cell survival/proliferation functions through regulation of a diverse array of genes [27–30]. Interestingly, despite the finding that RABV P-protein encodes STAT3 antagonist function [22], a recent study identified STAT3 as a pro-RABV factor, since RABV infection induced STAT3 phosphorylation, and suppression of STAT3 activity by pharmacological inhibition or siRNA knockdown reduced viral replication [31]. Thus, STAT3 appears likely to have both anti- and pro-viral functions during RABV infection. In common with other STATs, STAT3 forms different transcription factor complexes including STAT3 homodimers and STAT3-STAT1 heterodimers. STAT3 homodimers and heterodimers are likely to enable regulation of distinct gene profiles, as has been suggested for such complexes of other STATs [32–35], and so could account, at least in part, for pro- and anti-viral roles of STAT3. However, despite major roles for STAT3 in human health, including in cancers, the signalling profiles and biological roles of specific STAT3 dimers remains poorly understood. Given the potential divergence in functions of different STAT3 dimers, an intriguing possibility is that selective targeting by viral antagonists might enable suppression of antiviral genes while favouring expression of other genes that support cellular conditions amenable to infection/spread. Furthermore, such targeting could provide new tools to probe the function of specific STAT3 pathways. These possibilities have not been investigated.

Here, we aimed to investigate the lyssavirus P-protein-STAT3 interaction, finding that P-protein uses a novel, dimer-selective strategy to block responses by STAT3-STAT1 heterodimers, but not STAT3 homodimers, and so can exert differential regulation of IL-6-activated genes. This provides insight into the nature of viral regulation of innate immunity, and of differential gene regulation by STAT complexes.

## Results

### STAT3 is antagonised by P-proteins of diverse lyssaviruses

Previous data indicate that the P-protein of RABV challenge virus standard (CVS) strain (CVS-P) can bind to and inhibit nuclear accumulation of STAT3 following cellular activation by the IL-6 family cytokine oncostatin M (OSM) or by IFN-α, and so suppress OSM-induced gene expression [22]. To examine whether this is conserved between lyssaviruses, we assessed STAT3 antagonism by a panel of P-proteins from viruses including the RABV fixed pathogenic strain Nishigahara (Nish-P) and Nish-derived attenuated strain Ni-CE; the highly pathogenic ‘street’ strain silver-haired bat rabies virus (SHBRV-P); and the lyssaviruses ABLV, MOKV, and DUVV. The attenuated Ni-CE strain is defective for IFN/STAT1 antagonism due to substitutions in the P gene that inhibit P-protein suppression of STAT1 signalling, including through deactivation of the N-NES, which disables P-protein-mediated nuclear exclusion of STAT1 [36]. These data indicated that the P-protein is a pathogenesis factor, dependent on its IFN-antagonist function, but effects on responses to other cytokines have not been reported.

**Fig 1:**
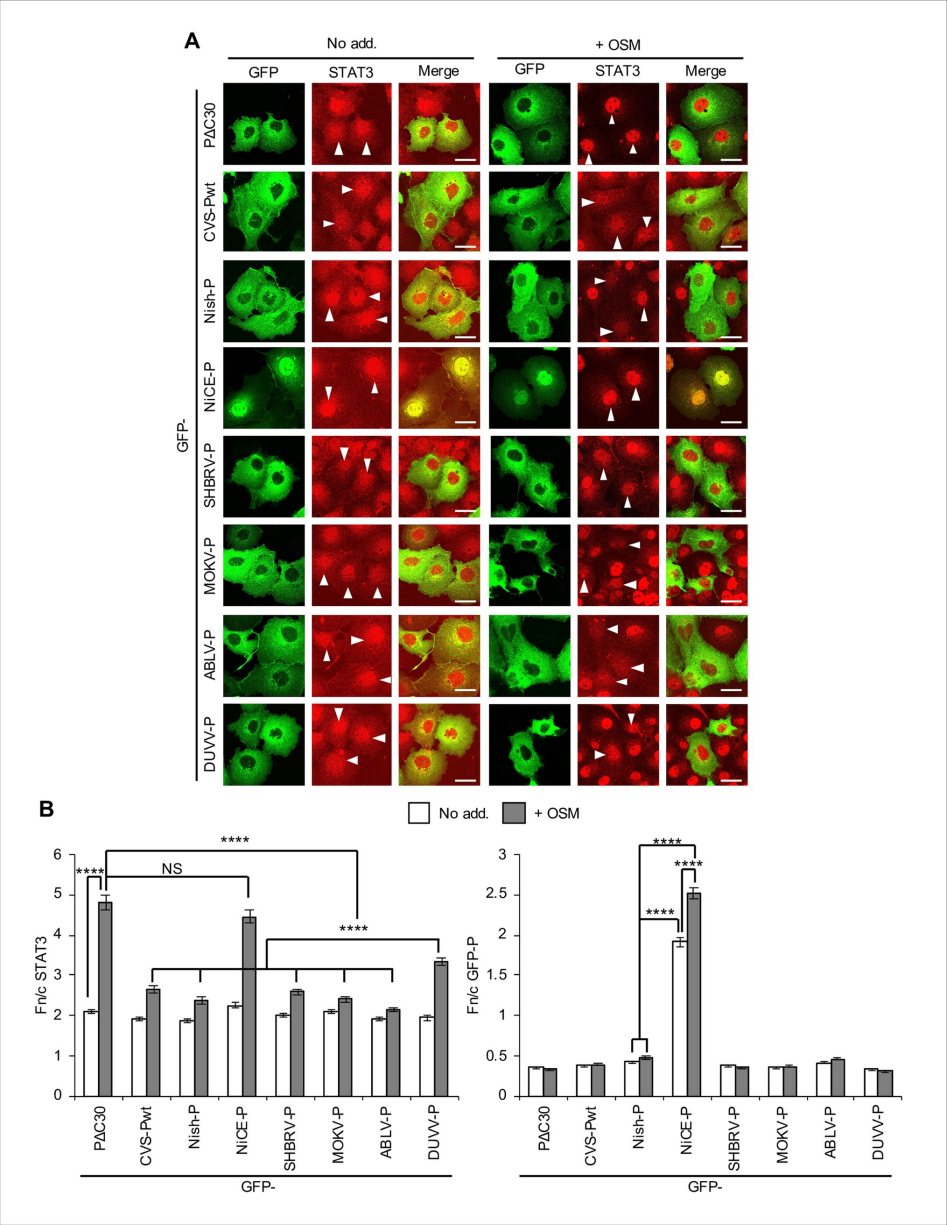
P-proteins from diverse lyssaviruses inhibit STAT3 responses to OSM. (A) COS7 cells transfected to express the indicated proteins were treated 24 h post-transfection with or without (No add.) OSM (10 ng/ml, 15 min) before fixation, immunofluorescent staining for STAT3 (red) and analysis by CLSM. Representative images are shown. Arrowheads indicate cells with detectable expression of the transfected protein. Scale bars, 30 μm. (B) Images such as those shown in A were analysed to calculate the ratio of nuclear to cytoplasmic fluorescence (Fn/c) for STAT3 (left panel) and P**-**proteins (right panel) in GFP-positive cells; histograms show mean ± standard error of the mean (SEM; n ≥ 47 cells for each condition; results are from a single assay representative of three independent assays). Statistical analysis used Student’s *t* test. ****, p < 0.0001; NS, not significant; No add., no addition. Cells lacking detectable expression of the transfected protein were also analysed to determine the Fn/c for STAT3 as an internal negative control (see S1 Fig).

COS7 cells expressing P-proteins fused to GFP were treated with OSM before immunofluorescent staining of STAT3 and analysis by confocal laser scanning microscopy (CLSM; Fig 1A) to quantify nucleocytoplasmic localisation of the proteins by calculating the nuclear to cytoplasmic fluorescence ratio (Fn/c, Fig 1B and S1 Fig) [5, 15, 22]. OSM induced clear nuclear accumulation (≥ 2 fold increase in Fn/c) of STAT3 in cells expressing PΔC30 (Fig 1B), a variant of CVS P-protein in which a deletion at the C-terminus prevents interaction with STAT1 or STAT3 [12, 22], and in cells lacking detectable expression of the transfected GFP proteins (S1 Fig). Full-length wildtype (wt) CVS-Pwt was clearly inhibitory (as previously shown [22]), and we confirmed STAT3 antagonist activity in cells treated with IL-6 or IFN-α (S2 Fig). Importantly, P-proteins of all RABV strains (except for the attenuated Ni-CE, see below) and lyssaviruses examined were also inhibitory (Fig 1A and 1B; S1 Fig); this included the distantly related MOKV, indicative of a conserved antagonistic strategy.

All P-proteins except Ni-CE-P localised strongly to the cytoplasm (Fig 1A), consistent with conserved activity of the N-NES [15, 37]. Ni-CE-P displayed more nuclear localisation and, importantly, did not inhibit OSM-induced STAT3 nuclear accumulation (Fig 1B; S1 Fig), similar to previous observations for IFN-activated STAT1 [5]. A significant increase in Ni-CE-P nuclear accumulation was also evident following OSM treatment, accompanying strong nuclear accumulation of STAT3, while OSM treatment had negligible effect on the localisation of other P-proteins (Fig 1B, right panel). Thus, Ni-CE-P appears to be partially relocated to the nucleus by a ‘piggyback’ mechanism due to association with OSM-activated STAT3 complexes and defective nuclear export; this is again consistent with observations for IFN-activated cells [5]. Thus, lyssavirus P-proteins appear to use a conserved N-NES-dependent strategy to actively export STAT1, 2 and 3 out of the nucleus, which enables antagonism of responses by different cytokine-dependent pathways. Importantly, the finding that Nish and Ni-CE P-proteins differ in antagonism of STAT3 indicates that the capacity to inhibit STAT3 correlates with pathogenicity [5, 38].

### Substitutions in the P-protein W-hole impair STAT3 antagonism

**Fig 2:**
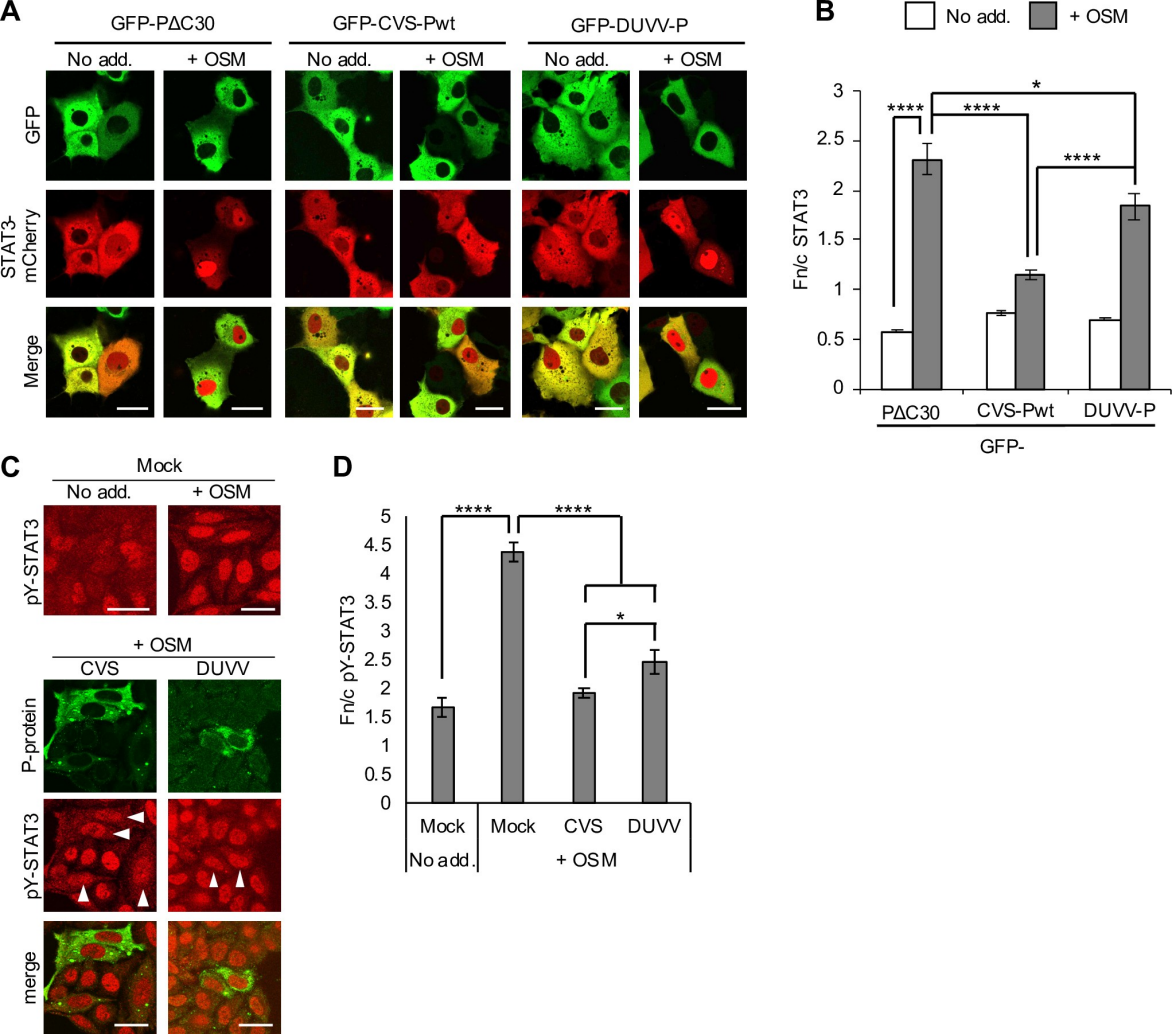
DUVV is defective for STAT3 antagonism compared with RABV. (A) COS7 cells co-transfected to express the indicated proteins were treated 24 h post-transfection with or without OSM (10 ng/ml, 30 min) before live-cell CLSM. (B) Images such as those shown in A were analysed to calculate the Fn/c for STAT3-mCherry (mean ± SEM; n ≥ 68 cells for each condition; results are from a single assay representative of two independent assays). (C,D) HeLa cells infected with CVS or DUVV (MOI of 1) or mock-infected were treated 48 h post-infection with or without OSM (10 ng/ml, 45 min) before fixation, staining for P-protein (green) and pY-STAT3 (red) and immunofluorescence analysis (C) to determine the Fn/c for pY-STAT3 (D; mean ± SEM, n ≥ 13 cells). Arrowheads indicate cells in which infection was clearly detectable. Scale bars, 30 μm. Statistical analysis used Student’s *t* test. *, p < 0.05; ****, p < 0.0001.

**Fig 3:**
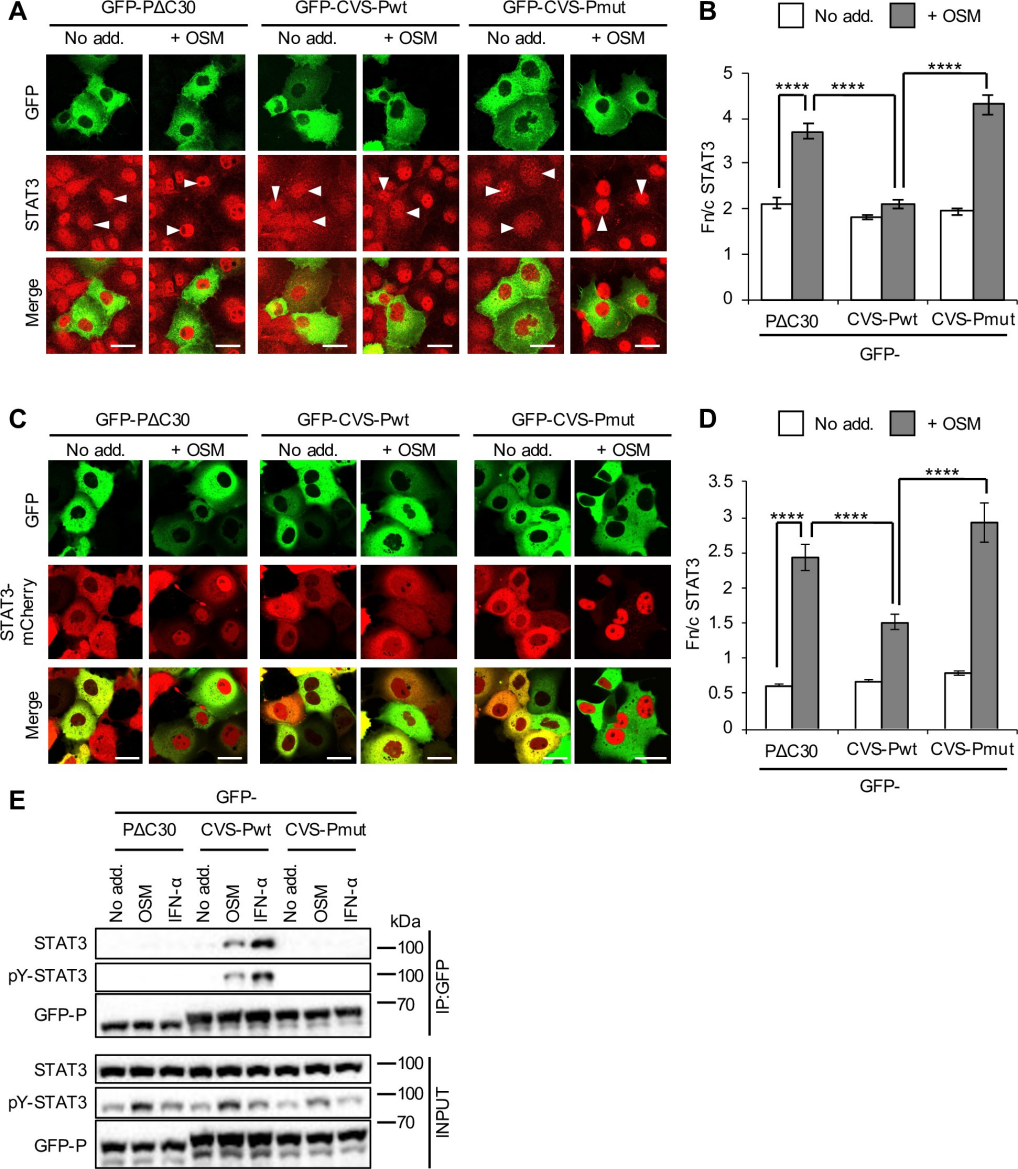
Mutation of RABV P-protein residues W265 and M287 prevents antagonism of STAT3. (A) COS7 cells transfected to express the indicated proteins were treated with or without OSM before immunofluorescent staining for STAT3 and analysis by CLSM, as described in the legend to Fig 1. Representative images are shown. Arrowheads indicate cells with detectable expression of the transfected protein. (B) Images such as those shown in A were analysed to calculate the Fn/c for STAT3 (mean ± SEM; n ≥ 31 cells for each condition; results are from a single assay representative of four independent assays). (C,D) COS7 cells co-transfected to express the indicated proteins were treated with or without OSM before live-cell CLSM (C) to determine the Fn/c for STAT3-mCherry (D; mean ± SEM; n ≥ 59 cells for each condition; results are from a single assay representative of two independent assays), as described in the legend to Fig 2. Scale bars, 30 μm. Statistical analysis used Student’s *t* test. ****, p < 0.0001. (E) HEK293T cells transfected to express the indicated proteins were treated 24 h post-transfection with or without OSM (10 ng/ml, 15 min) or IFN-α (1,000 U/ml, 30 min) before lysis and immunoprecipitation for GFP. Lysates (input) and immunoprecipitates (IP) were analysed by immunoblotting (IB) using antibodies against the indicated proteins. Results are representative of 3 independent assays.

Although DUVV-P is inhibitory to STAT3, this activity is significantly reduced compared with other antagonistic P-proteins examined in immunostained cells (Fig 1B, left panel) and living cells co-expressing mCherry-fused STAT3 (Fig 2A and 2B). Analysis of cells infected with RABV and DUVV confirmed a conserved capacity to inhibit STAT3 nuclear accumulation, with the activity of DUVV modestly but significantly reduced (Fig 2C and 2D). DUVV-P was previously shown to be defective for binding to STAT1 compared with P-proteins of other lyssaviruses, which appears to relate to a substitution in the ‘W-hole’ hydrophobic pocket in the P-protein CTD (W265 in RABV P-protein for G265 in DUVV P-protein) [6]. Combined substitution of two residues (W265 to G and M287 to V; W265G/M287V) in the W-hole of RABV or DUVV P-proteins strongly impairs IFN/STAT1 binding/antagonism and pathogenesis of recombinant RABV [6]. Notably, analysis by CLSM of immunostained and living cells expressing CVS P-protein containing W265G/M287V substitutions (CVS-Pmut) revealed clear loss of antagonism toward OSM-induced STAT3 nuclear accumulation (Fig 3A–3D). Analysis of immunoprecipitates of GFP-P-proteins from HEK293T cells (Fig 3E) indicated that CVS-Pwt clearly co-precipitates STAT3 from OSM and IFN-α-treated cells, with efficient co-precipitation requiring activation/phosphorylation of STAT3, as expected [22]. PΔC30 and CVS-Pmut were strongly impaired for interaction. These data indicate that STAT3 is bound by the P-protein CTD in an analogous fashion to STAT1, and likely involves the same or overlapping regions.

### Antagonism of OSM-activated genes differs between RABV P-protein and mumps virus V-protein

The above data suggested two likely possibilities. In the first, P-protein binds to a conserved/homologous site on STAT1 and STAT3 to directly antagonise both proteins, a reasonable scenario given the high degree of similarity in STAT1/3 domain structure and sequence [39] (72% protein sequence similarity). Such independent targeting of STAT1 and STAT3 was reported for mumps virus (MUV) V-protein, which induces degradation of both proteins [16, 40]. Alternatively, RABV P-protein CTD directly targets STAT1 (as confirmed recently using biophysical techniques [8]), but does not specifically contact STAT3 and rather interacts with STAT3 in the context of activated STAT3-STAT1 heterodimers. The latter is intriguing as it could result in selective antagonism of IL-6-activated pY-STAT3-STAT1 heterodimers without impacting on distinct pY-STAT3 complexes such as STAT3 homodimers, potentially resulting in selective gene regulation. Interestingly, despite more potent activation of STAT3 by OSM compared with IFN-α (see pY-STAT3 levels in input, Fig 3E), CVS-Pwt clearly co-precipitated more STAT3 in response to IFN-α than OSM. IFN-α is a potent activator of STAT1, and we confirmed that IFN-α treatment induced higher levels of pY-STAT1 than OSM, in direct contrast to induction of pY-STAT3 (S3 Fig). These data suggested that IFN-α will induce higher levels of STAT3-STAT1 heterodimers than OSM; thus, the greater co-precipitation of STAT3 with P-protein from IFN-α-activated cells would appear consistent with targeting *via* activated STAT1.

**Fig 4:**
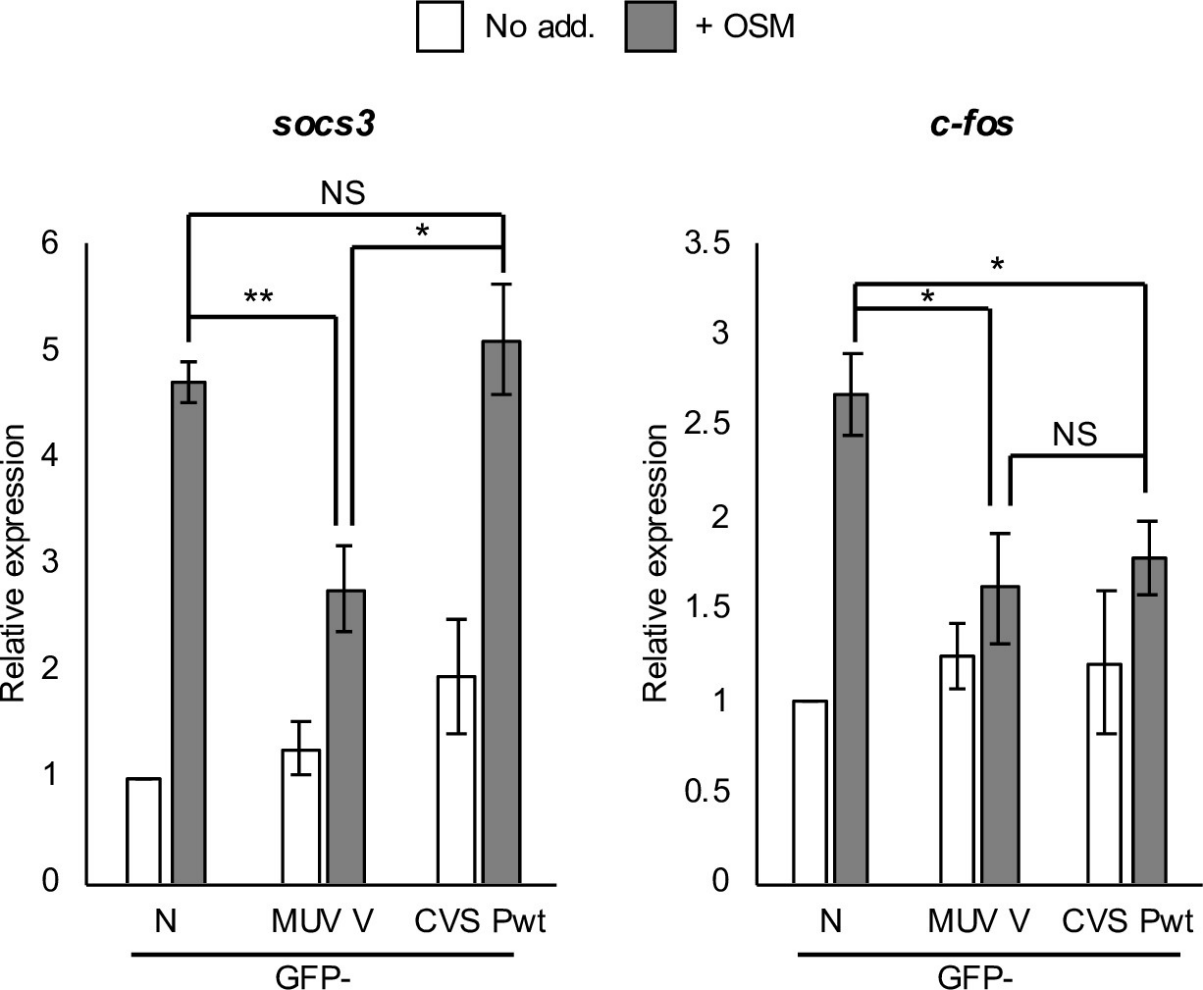
RABV P-protein and MUV V-protein differentially regulate OSM/STAT3-activated target genes. HEK293T cells transfected to express the indicated proteins were treated 24 h post-transfection with or without OSM (10 ng/ml, 45 min for *soc3*, 60 min for *c-fos*) before analysis by RT-qPCR. Histograms show expression of *socs3* and *c-fos* normalised to *gapdh* and calculated relative to that determined for untreated GFP-N-protein-expressing cells (mean ± SEM; n = 3 independent assays). GFP-N, GFP-RABV CVS nucleoprotein; *, p < 0.05; **, p < 0.01; NS, not significant.

The specific roles of STAT3 heterodimers and homodimers in regulating IL-6 cytokine-inducible genes are poorly understood. However, maximal induction of the STAT3 target gene *c-fos* in response to OSM was found to be dependent on the expression of both STAT3 and STAT1 [41]. Furthermore, electrophoretic mobility shift assays (EMSA) indicated that IL-6 induces substantial binding of STAT3 homodimers and STAT3-STAT1 heterodimers to the m67 probe, which is a variant of a STAT-binding sequence derived from the *c-fos* promoter [42–44]. In contrast, EMSA analyses indicated that the promoter of *socs3*, a well-established IL-6/STAT3 target gene, appears to be predominantly bound by STAT3 homodimers in response to IL-6 or the IL-6 family cytokine leukaemia inhibitory factor [45, 46]. We reasoned that these genes might be induced to differing extents by STAT3-STAT1 and STAT3-STAT3 complexes, with *socs3* largely affected by the latter. Thus, MUV V-protein would be expected to inhibit OSM-dependent *socs3* expression, but RABV P-protein might differ, depending on whether it binds STAT3 directly or only in the context of STAT3-STAT1 complexes. We thus performed RT-qPCR analysis of *socs3* expression, confirming substantial upregulation by OSM, which was clearly inhibited by MUV V-protein (Fig 4). However, RABV P-protein had no inhibitory effect across multiple assays. In contrast, P-protein was able to suppress *c-fos* activation by OSM (Fig 4). These data indicated that RABV P-protein and MUV V-protein differ in their capacity to target specific genes, possibly due to distinct targeting of STAT3 dimers by P-protein.

### RABV P-protein-STAT3 interaction is dependent on STAT1

**Fig 5:**
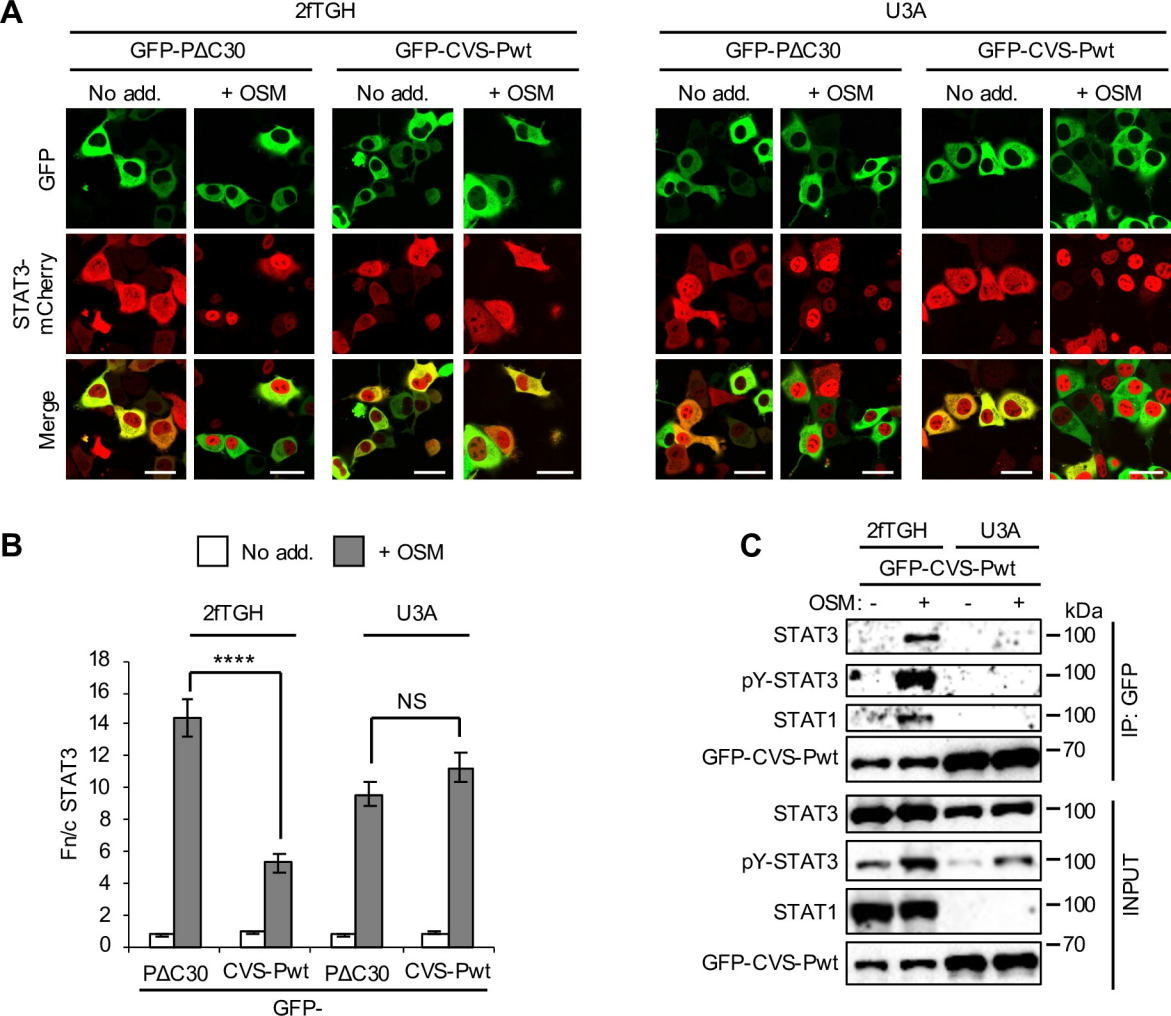
RABV P-protein does not interact with STAT3 in STAT1-deficient U3A cells. (A,B) 2fTGH and U3A cells co-transfected to express the indicated proteins were treated with or without OSM before live-cell CLSM (A) to determine the Fn/c for STAT3-mCherry (B; mean ± SEM; n > 71 cells for each condition; results are from a single assay representative of two independent assays), as described in the legend to Fig 2. Scale bars, 30 μm. Statistical analysis used Student’s *t* test. ****, p < 0.0001; NS, not significant. (C) 2fTGH and U3A cells transfected to express GFP-CVS-Pwt were treated with or without OSM before immunoprecipitation of GFP and analysis by IB using antibodies against the indicated proteins, as described in the legend to Fig 3. Results are representative of 3 independent assays.

**Fig 6:**
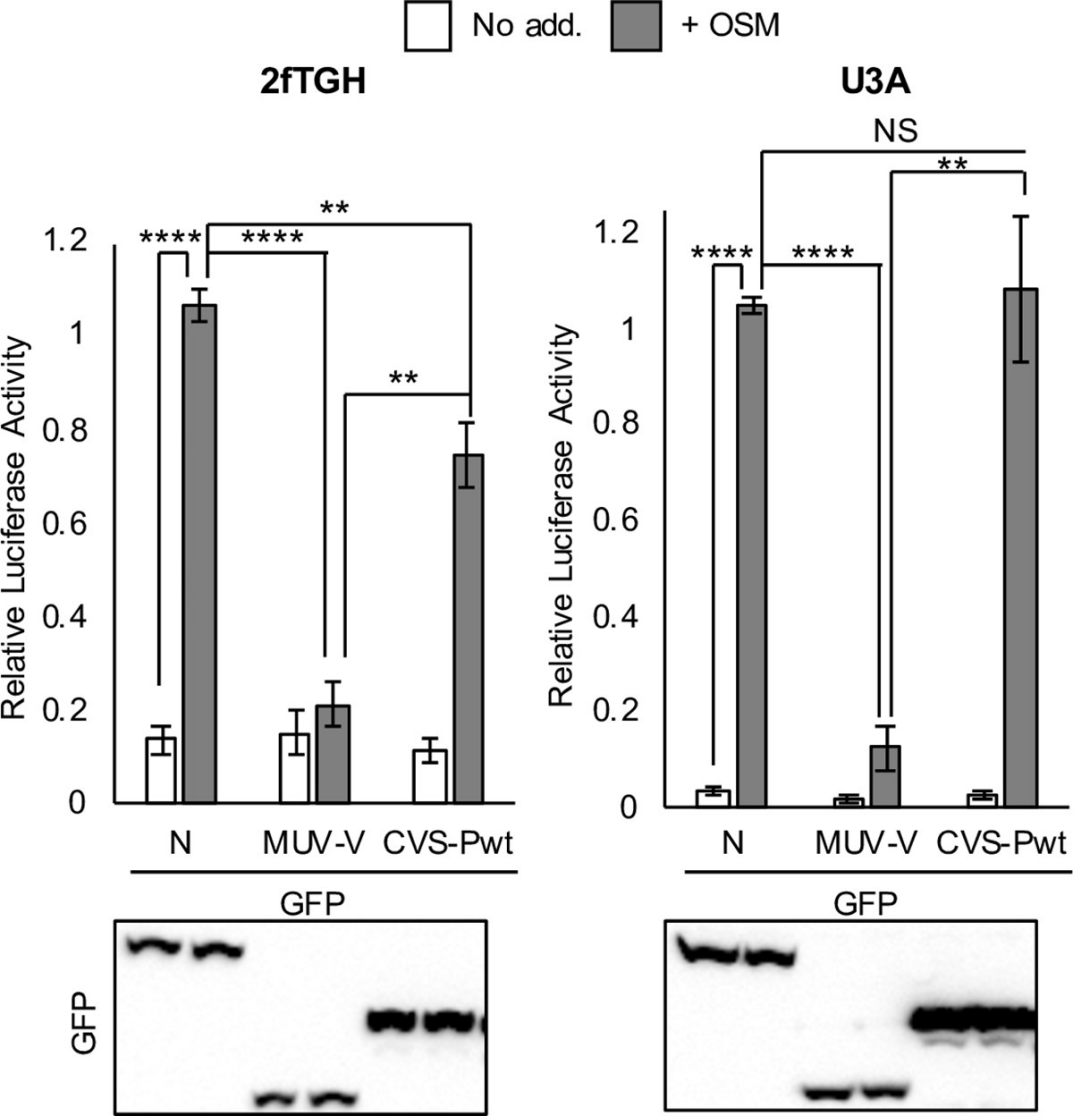
Antagonism of STAT3 by RABV P-protein is defective in STAT1-deficient U3A cells. 2fTGH or U3A cells co-transfected with m67-LUC and pRL-TK plasmids, and plasmids to express the indicated proteins, were treated 16 h post-transfection with or without OSM (10 ng/ml, 8 h) before determination of relative luciferase activity (mean ± SEM; n = 3 independent assays, upper panel); *lower panel*: cell lysates used in a representative assay were analysed by IB for GFP. Statistical analysis used Student’s *t* test. **, p < 0.01; ****, p < 0.0001; NS, not significant.

To directly examine the role of STAT3-STAT1 complexes in STAT3 targeting by P-protein, we used 2fTGH cells and a 2fTGH-derivative STAT1-null line, U3A, which have been used extensively to study STAT1 function (eg. [47, 48]). CLSM analysis of living cells expressing STAT3-mCherry indicated that CVS-Pwt strongly inhibits OSM-induced STAT3 responses in 2fTGH but has no effect in U3A cells (Fig 5A and 5B). Consistent with this, immunoprecipitation assays indicated that OSM-activated STAT3 and STAT1 co-precipitate with CVS-Pwt from 2fTGH but not U3A cells (Fig 5C; S4 Fig). Furthermore, reporter gene assays for IL-6-induced gene expression (using the m67-luciferase plasmid, as previously [22]) indicated that CVS-Pwt effects significant inhibition of OSM signalling in 2fTGH cells but not U3A cells (Fig 6). Similar inhibition was observed in HEK293T cells, which also express STAT1 (S5 Fig). Thus it appears that P-protein-STAT3 interaction requires STAT1, consistent with selective targeting of STAT3-STAT1 complexes. In contrast, MUV V-protein strongly inhibited luciferase induction in all cell types (Fig 6; S5 Fig), consistent with independent targeting of STAT3 and STAT1.

Of note, these assays indicated that the extent of antagonism in STAT1-positive cells differs between RABV P-protein and MUV V-protein (Fig 6; S5 Fig), possibly relating to differential targeting of STAT complexes. This difference observed in the reporter assays was not clearly evident in RT-qPCR analysis of *c-fos* expression (Fig 4). This is perhaps due to differences in the nature of the assays, where the former detects protein expression 8 h post-OSM treatment from plasmid carrying m67 (an enhanced variant of a minimal STAT-binding sequence of the *c-fos* promoter [44]), and the latter detects transcript induced from the endogenous *c-fos* gene soon after treatment; thus, the extent and kinetics of promoter interaction with STAT dimers might differ. However, the data together clearly indicate that P-protein inhibits OSM-activated gene expression dependent on STAT1 (Fig 6), and differentially impacts expression of specific endogenous target genes (Fig 4).

Importantly, the capacity of an IFN-antagonist to bind STAT1 does not *per se* confer binding to STAT3, as reported for Hendra virus (HENV) V-protein [24], which binds STAT1 and STAT2 independently of tyrosine phosphorylation and inhibits nuclear translocation in response to IFN, but does not co-precipitate with STAT3 from non-activated cells [24]. To confirm the lack of STAT3 interaction in cytokine-activated cells, we assessed OSM-activated STAT3 nuclear localisation in COS7 cells, finding the HENV V-protein, which is exclusively cytoplasmic, has no inhibitory effect, in contrast to MUV V-protein and RABV P-protein (S6 Fig). We saw consistent results in HeLa cells (S6 Fig), indicating that the lack of STAT3 targeting is not due to host species/cell type. Thus, STAT1-dependent targeting of STAT3 by RABV P-protein represents a specific mechanism.

**Fig 7:**
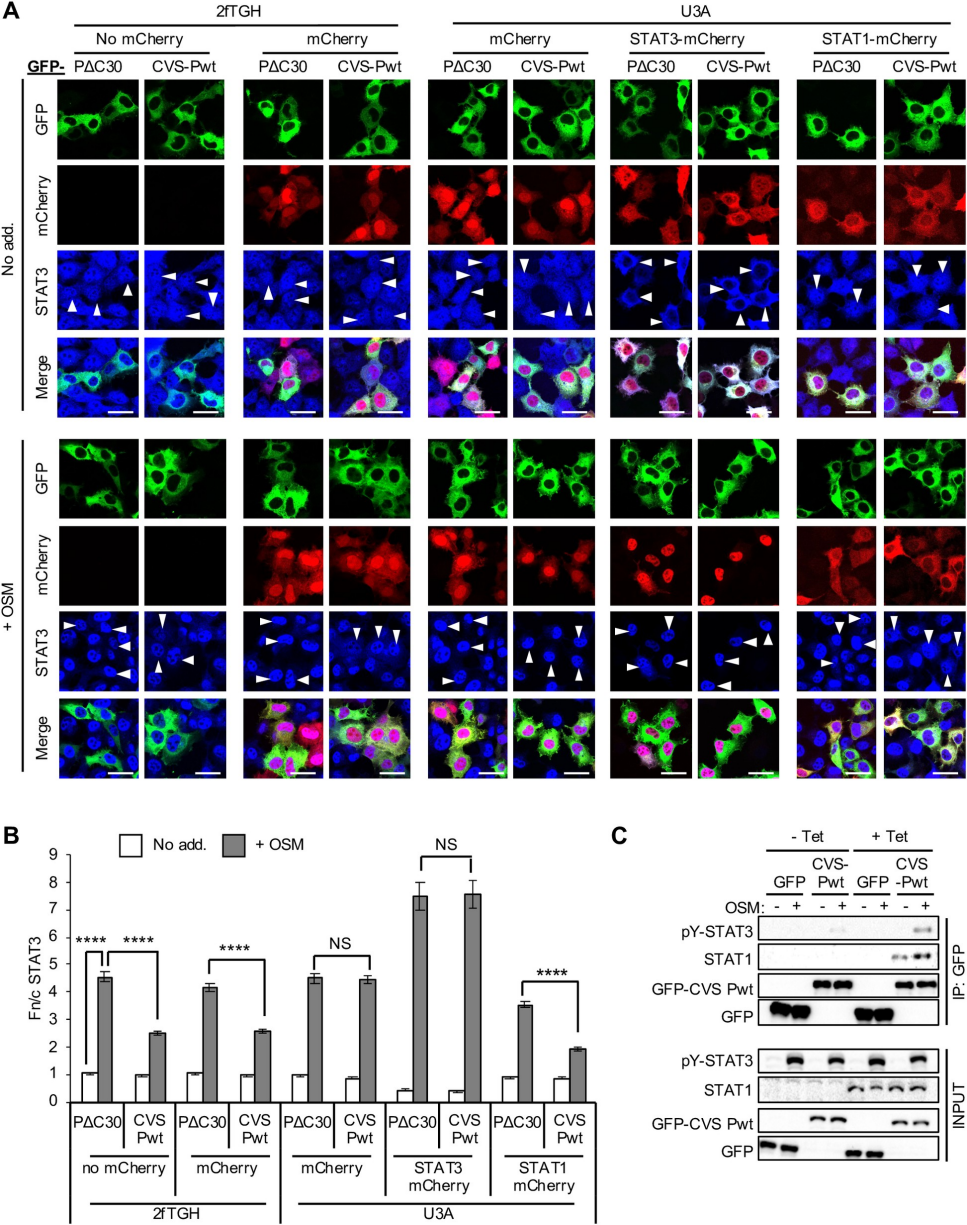
RABV P-protein antagonism of STAT3 is restored in U3A cells by STAT1 expression. (A) 2fTGH and U3A cells co-transfected to express the indicated proteins were treated with or without OSM (10 ng/ml, 30 min) before fixation, immunofluorescent staining for STAT3 (blue, Alexa Fluor 647) and analysis by CLSM, as described in the legend to Fig 1. Representative images are shown. Arrowheads indicate cells with detectable expression of transfected proteins. Scale bars, 30 μm. (B) Images such as those shown in A were analysed to calculate the Fn/c for STAT3 (mean ± SEM; n ≥ 40 cells for each condition; results are from a single assay representative of three independent assays). Statistical analysis used Student’s *t* test. ****, p < 0.0001; NS, not significant. (C) U3A cells stably transfected with plasmid for tetracycline-inducible expression of STAT1 were cultured with or without 1 μg/ml tetracycline (Tet) to induce STAT1 expression and transfected to express GFP or GFP-CVS-Pwt. Cells were treated 24 h post-transfection with or without OSM before immunoprecipitation of GFP and analysis by IB, as described in the legend to Fig 3. Results are representative of two independent assays.

To confirm directly the dependence of STAT3 antagonism by P-protein on STAT1, we reconstituted STAT1 expression in U3A cells by co-transfection to express mCherry-STAT1 or expression controls (mCherry alone or mCherry-STAT3). CLSM analysis of cells immunostained for STAT3 indicated that STAT1 expression is sufficient to recapitulate antagonism of OSM-activated STAT3 by CVS-Pwt, with the extent of STAT3 inhibition comparable to that in 2fTGH cells (Fig 7A and 7B). Importantly, we saw no comparable effect of STAT3 overexpression. Thus, the effect of STAT1 expression is not simply due to increased cellular levels of STAT complexes, with the data directly supporting the idea that P-protein targets STAT3-STAT1 heterodimers, but not STAT3 homodimers.

**Fig 8:**
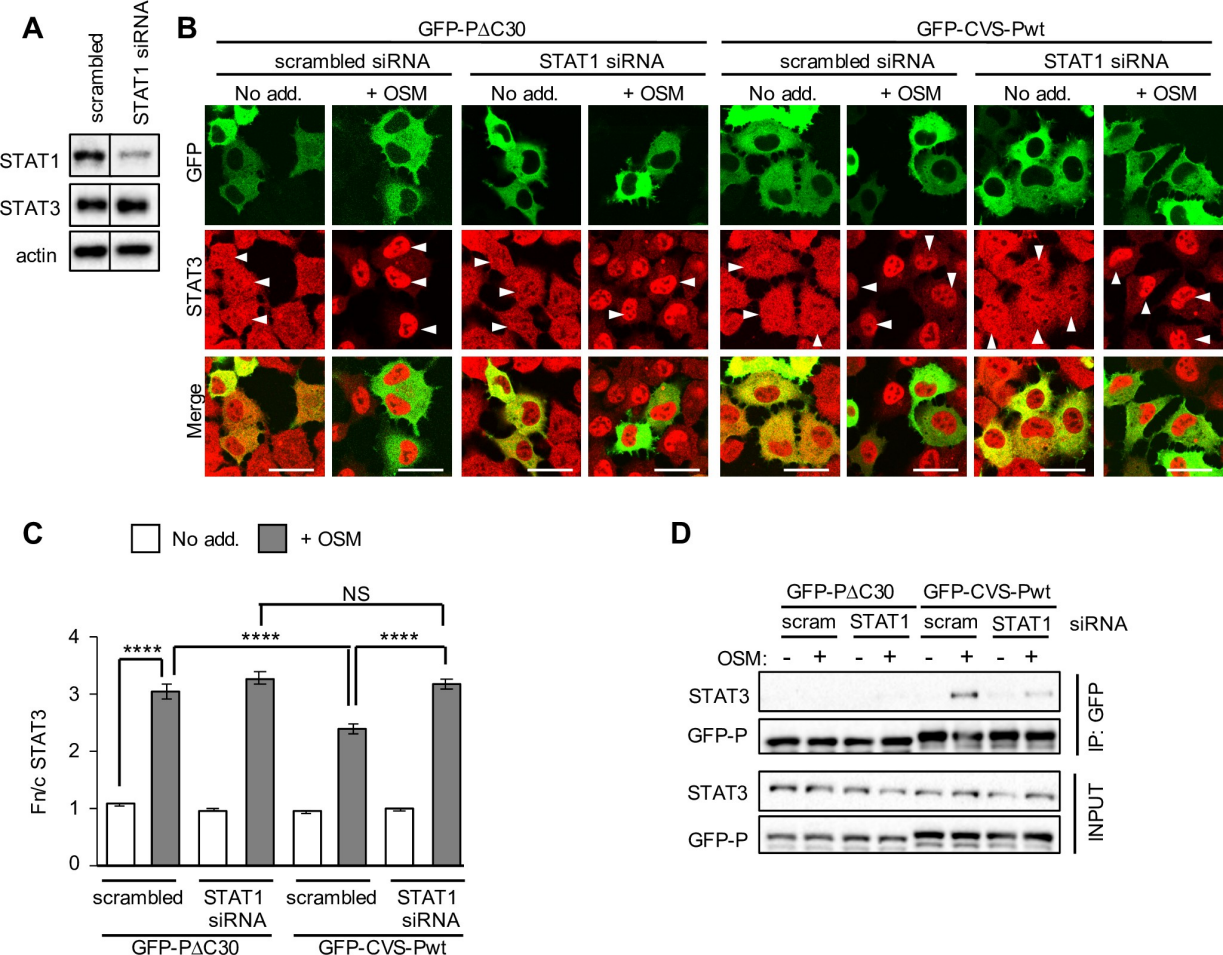
RABV P-protein antagonism of STAT3 is impaired by depletion of STAT1. (A) 2fTGH cells were transfected with 100 nM scrambled siRNA or siRNA specific for STAT1 (STAT1 siRNA) for 48 h before lysis and IB analysis using antibodies against the indicated proteins. Actin was used as loading control. (B) 2fTGH cells were transfected with siRNA (as described in A) for 24 h before transfection to express the indicated protein for a further 24 h. Cells were then treated with or without OSM before immunofluorescent staining for STAT3 (red) and analysis by CLSM as described in the legend to Fig 1. Representative images are shown. Arrowheads indicate cells with detectable expression of the transfected protein. Scale bars, 30 μm. (C) Images such as those shown in B were analysed to calculate the Fn/c for STAT3 (mean ± SEM; n ≥ 55 cells for each condition; results are from a single assay representative of two independent assays). Statistical analysis used Student’s *t* test. ****, p < 0.0001; NS, not significant. (D) 2fTGH cells transfected with siRNA and plasmids to express the indicated proteins (as described in B) were treated with or without OSM before immunoprecipitation of GFP and analysis by IB as described in the legend to Fig 3. Results are representative of four independent assays.

We further analysed P-protein-STAT3 interaction by immunoprecipitation using a tetracycline-inducible stable transfection system to express non-fused STAT1 in U3A cells, confirming that STAT1 expression clearly enhanced co-precipitation of pY-STAT3 with CVS-Pwt (Fig 7C). Finally, knockdown of STAT1 in 2fTGH cells using siRNA (Fig 8A) impaired antagonistic activity of CVS-Pwt toward OSM-activated STAT3 nuclear accumulation (Fig 8B and 8C). STAT1 knockdown also clearly reduced OSM-dependent interaction of CVS-Pwt with STAT3 (Fig 8D); although the loss of STAT3 interaction was not complete, the observed reduction of interaction is consistent with the observed knockdown of STAT1 (Fig 8A). Together these data using U3A and 2fTGH, and different methods to reduce or increase expression of STATs, indicate that the lack of STAT3 targeting by P-protein in U3A is due to the absence of STAT1, rather than other parameters such as differing basal STAT3 expression levels. Thus, P-protein requires STAT1 to antagonise STAT3, consistent with selective targeting of STAT1 and, thereby, STAT3-STAT1 heterodimers, but not STAT3 homodimers.

## Discussion

Here we have shown that antagonism of STAT3 is conserved among P-proteins from diverse, highly lethal lyssaviruses, including the RABV street strain SHBRV and the closely related ABLV (RABV and ABLV are members of *Lyssavirus* phylogroup I), as well as the distantly related MOKV of phylogroup II, indicative of conserved roles in lyssavirus infection. Thus, lyssaviruses form part of a limited cohort of negative-sense RNA viruses shown to target STAT3, which includes the paramyxoviruses MUV, measles and Tioman virus [16–18]. Together with reports of STAT3 antagonism by more diverse viruses including double-stranded DNA [19] and positive-sense RNA viruses [20], these data suggest that STAT3 targeting has important and likely highly specific roles in the biology of certain viruses. This likely involves antagonism of signalling by a broad array of cytokines that signal via STAT3, including IL-6 cytokine family members (e.g. IL-6, leukaemia inhibitory factor and ciliary neurotrophic factor), IFNs, IL-10, IL-2, IL-22, granulocyte-macrophage colony-stimulating factor and epidermal growth factor [26]. Selective evasion of multiple cytokine pathways may be of particular importance to lyssaviruses due to their broad host range, infection of multiple organs/cell types and often lengthy incubation [1, 4, 49].

We found that the efficiency of targeting/antagonism of STAT3 differs between specific lyssaviruses and attenuated RABV, correlating with differing targeting efficiency toward STAT1 [5, 6], and that this is due to the interaction with STAT3 being mediated indirectly *via* STAT1 in the context of STAT3-STAT1 heterodimers. Importantly, targeting of STAT1 does not necessarily confer to IFN-antagonists the capacity to interact with STAT3, as shown here and previously for V-proteins of paramyxoviruses HENV, Nipah virus and simian virus 5 (S6 Fig) [16, 24]. Thus, it appears that P-protein specifically inhibits STAT3-STAT1 heterodimers but not STAT3 homodimers such that signalling by the latter would be unimpaired. In fact, although CVS-Pwt clearly and consistently suppressed OSM signalling leading to luciferase expression from the m67-LUC plasmid in 2fTGH cells (and HEK293T cells), its effect was partial compared with MUV-V, which resulted in signalling approaching or equivalent to that in non-treated cells (Fig 6 and S5 Fig). This is consistent with a global shut-down of STAT3 signalling by MUV V-protein compared with targeting of a subset of signalling complexes (i.e. STAT3-STAT1 heterodimers) by P-protein. Indeed, RT-qPCR analysis indicated that P-protein selectively regulates gene expression in response to OSM, being able to inhibit *c-fos* but not *socs3* expression.

The specific biological roles of STAT3-STAT1 heterodimers and most other STAT heterodimers are poorly understood [32]. Perhaps the best characterised STAT heterodimer is STAT1-STAT2, which is activated by type I IFNs and, in complex with IFN regulatory factor 9, binds to IFN-stimulated response elements within IFN-stimulated gene (ISG) promoters to activate transcription. In contrast, STAT1 homodimers, which are activated by both type I and II IFNs, bind to gamma-activated sequences (GAS) within a distinct but overlapping set of ISGs. STAT1-STAT2 heterodimers are therefore a major determinant of the distinct outcomes of type I and type II IFN signalling [33]. Direct evidence for functional differences of other pairs of STAT homo/heterodimers is very limited. A ChIP-reChIP analysis found that the *ebi3* and *il12a* promoters are strongly bound by IL-35-activated STAT1-STAT4 heterodimers but not by STAT1 or STAT4 homodimers (activated by IFN-γ or IL-12, respectively) [34]. Consistent with this, expression of both STAT1 and STAT4 was required for maximal induction of *ebi3* and *il12a* by IL-35. Importantly, the STAT1-STAT4 heterodimer did not bind to the promoters of classical STAT1 and STAT4 target genes *irf1* and *il18ra*, respectively; together, these data suggest that STAT1-STAT4 complexes likely regulate gene profiles distinct from those regulated by STAT1 or STAT4 homodimers [34]. Similarly, EMSA analysis of STAT-DNA complexes formed in response to colony stimulating factor 1 and platelet-derived growth factor found that STAT3-STAT5 heterodimers interacted with the m67 probe but not the prolactin-inducible element from the bovine casein gene promoter, which was bound by STAT5 homodimers [35].

Clear evidence for gene-specific binding or regulation is lacking for STAT3-STAT1 complexes. While there are contrasting reports on whether STAT3 plays a positive or negative role in IFN signalling [50–59], one study proposed that STAT3 sequesters STAT1 in STAT3-STAT1 heterodimers and thereby reduces the amount of STAT1 homodimers bound to DNA in response to IFN-α, inhibiting the activation of certain GAS-containing ISGs (*irf1*, *cxcl9* and *cxcl10*) [55]. This implies that STAT3-STAT1 heterodimers may not be as effective at inducing these ISGs as STAT1 homodimers [55]. Nevertheless, our data using viral proteins specific for different STAT3 dimers is the first to indicate that STAT3-STAT1 heterodimers and STAT3 homodimers differentially contribute to gene expression. Specifically, the RT-qPCR assays indicated that OSM-induced *socs3* expression is suppressed by MUV V-protein but is unaffected by RABV P-protein, and thus likely to be largely induced by STAT3 homodimers. In contrast, the m67 luciferase and *c-fos* RT-qPCR assays indicate that *c-fos* expression is mediated, at least in part, by STAT3-STAT1 heterodimers, as it is inhibited by P-protein. Together these data support the idea that STAT heterodimers provide specific mechanisms to regulate particular functional genes and gene subsets, enabling fine-tuning of transcription and cellular responses; the formation of heterodimers, and hence functional outcomes, are likely influenced by factors including the integration of pathways activated by different cytokines acting on a cell at a given time, the expression levels of specific STAT members, and the type of cell. Moreover, the data support the utility of viral proteins that selectively target specific STATs/STAT complexes, such as HENV and MUV V-proteins, and RABV P-protein, as tools to delineate specific STAT signalling pathways.

While the full extent of the distinct functions of STAT3-STAT1 and STAT3-STAT3 dimers await further research, the biological impact of dimer selective targeting by P-protein is likely to be complex. STAT3 is implicated in antiviral immunity [50–52, 60, 61], inflammation [62, 63], cell survival and proliferation [64–66], and in regulating mitochondrial activity [67], autophagy [68], and microtubule stability [69, 70]. Because of this, global shutdown of STAT3 by virus is likely not only to impact antiviral pathways, but also cellular homeostasis more generally, impacting viability. This is likely to be more readily tolerated, or even beneficial for viruses with rapid replication cycles, but would be detrimental to lyssavirus infection that can have long incubation periods and relies on intact neuronal networks for viral entry and egress of the central nervous system [71]. Indeed, a lack of neuropathological damage indicative of “stealthy” infection of cells is a hallmark of RABV-infected brain samples [49, 72–74]. Selective targeting of activated (phosphorylated) STATs ([13, 22] and this study), combined with specificity for particular complexes is likely to support this, preventing ‘wholesale’ shutdown characteristic of some viruses, but enabling inhibition of induced antiviral mechanisms (mediated by STAT1/2 complexes and potentially STAT3-STAT1 complexes), without impacting homeostasis (potentially requiring STAT3 homodimers). The idea of a pro-RABV role for STAT3 complexes (likely STAT3 homodimers) is supported by recent data indicating that inhibition of STAT3 activity can impair RABV replication [31].

The great diversity in IFN-antagonist mechanisms used by viruses is likely to derive from specifics of the life cycle, including the duration of incubation and replication cycles, and cell tropism, such that closely related viruses including RABV and vesicular stomatitis virus use very different strategies [75]. The evolution of selective STAT targeting likely has similar evolutionary sources, with our data indicating selectivity not only between STAT family members, but also the dimers formed. Systems genomic analysis of the effects of P-protein on global cytokine/STAT3-dependent gene expression should expand our understanding of how this selective targeting modifies cellular functions, as well as the precise mechanisms of specific gene regulation by STAT3 complexes, a fundamental yet poorly understood aspect of cell biology important to immunity, development and cancer. Such knowledge would be hoped to contribute to efforts to develop novel viral vaccines and, potentially, antivirals, as well as approaches for STAT3-related pathologies such as cancer.

## Materials and methods

### Plasmids and cell culture

The construct to express MUV-V fused to GFP was generated by PCR amplification from MUV V-FLAG (a gift from Curt Horvath [16], Addgene plasmid #44908) and cloning into the pEGFP-C1 vector C-terminal to GFP (Clontech). STAT1 expression constructs were generated by PCR amplification from STAT1 plasmid and cloning into the pmCherry-N3 vector or the tetracycline-inducible expression vector pEBTetD (a gift from Dirk Gründemann, University of Cologne [76]). The construct to express mCherry-fused STAT3 was a kind gift from Marie Bogoyevitch (University of Melbourne). Other constructs have been described elsewhere [6, 15, 22].

2fTGH and U3A cells were a kind gift from George Stark, Lerner Research Institute, Cleveland Clinic. 2fTGH, U3A, COS7, HeLa and HEK293T cells were maintained in DMEM supplemented with 10% FCS and GlutaMAX (Life Technologies), 5% CO_2_, 37°C. Plasmid transfections used Lipofectamine 2000 (Invitrogen) or FuGene HD (Promega), according to the manufacturer’s instructions. siRNA (100 nM) was reverse-transfected into 2fTGH cells using Lipofectamine 2000 (Invitrogen), according to the manufacturer’s instructions. U3A cells stably transfected with pEBTetD-STAT1 were cultured with 1 μg/ml tetracycline to induce STAT1 expression.

### Microscopy

For analysis of STAT3 and P-protein localisation, cells growing on coverslips transfected with plasmids or infected with RABV (strain CVS; MOI of 1) or DUVV (strain 86132SA; MOI of 1) were incubated in serum-free-(SF)-DMEM for 1 h and treated without or with 10 ng/mL recombinant human OSM (BioVision) for 15 min (analysis of endogenous STAT3 in immunostained transfected cells), 30 min (analysis of STAT3-mCherry in living or immunostained transfected cells) or 45 min (analysis of infected cells). Cells were then fixed using 3.7% formaldehyde (10 min, room temperature, RT) followed by 90% methanol (5 min, RT) for transfected cells or cold methanol followed by 80% acetone for infected cells and immunostained. Antibodies used were: anti-STAT3 (Santa Cruz, sc-482), anti-pY-STAT3 (Cell Signaling Technology, 9145), anti-P-protein [77] and Alexa Fluor 488, 568 and 647 secondary antibodies (ThermoFisher Scientific).

Transfected cells were analysed by CLSM using a Leica SP5 or Nikon C1 Inverted confocal microscope with 63 X objective. For live cell analysis, cells were imaged in phenol-free DMEM using a heated chamber. Infected cells were analysed using a Zeiss Axioplan (version 2.2) fluorescence microscope equipped with a Zeiss ApoTome system, as previously [15]. Digitised confocal images were processed using Fiji software (NIH). To quantify nucleocytoplasmic localisation, the ratio of nuclear to cytoplasmic fluorescence, corrected for background fluorescence (Fn/c), was calculated for individual cells [15, 22].

### Co-immunoprecipitation

Transfected cells were incubated in SF-DMEM (3 h) before treatment with or without OSM (10 ng/ml, 15 min) or IFN-α (Universal Type I IFN, PBL Assay Science, 1000 U/ml, 30 min), lysis and immunoprecipitation using GFP-Trap Agarose beads (Chromotek), according to the manufacturer’s instructions. Lysis and wash buffers were supplemented with PhosSTOP (Roche), cOmplete Protease Inhibitor Cocktail (Roche) and 10 mM NaF. Lysates and immunoprecipitates were analysed by SDS-PAGE and immunoblotting (IB) using antibodies against STAT3 (above), pY-STAT3 (above), STAT1 (BD Biosciences, 610119 or 610185), pY-STAT1 (Cell Signaling Technology, 9167 or Santa Cruz, sc-7988), GFP (Roche Applied Science, 11814460001), actin (Abcam, ab3280) and HRP-conjugated secondary antibodies (Merck). Visualisation of bands used Western Lightning chemiluminescence reagents (PerkinElmer).

### Luciferase reporter gene assays

Cells were co-transfected with Firefly luciferase plasmid m67-LUC and Renilla luciferase plasmid pRL-TK, as previously described [22], and protein expression constructs for 16 h. Cells were then treated without or with OSM (10 ng/mL, 8 h) before lysis using Passive Lysis Buffer (Promega). Firefly and Renilla luciferase activity was determined in a dual luciferase assay, as previously described [6, 22]. The ratio of Firefly to Renilla luciferase activity was determined for each condition, and then calculated relative to that determined for GFP-N-protein-expressing cells treated with OSM (relative luciferase activity). Data from ≥ 3 independent assays were combined, where each assay result is the mean of three replicate samples.

### RT-qPCR

Transfected HEK293T cells were incubated in SF-DMEM (3 h) before treatment without or with OSM (10 ng/ml, 45 min for *socs3*, 60 min for *c-fos*) and RNA extraction (ReliaPrep RNA Cell Miniprep System, Promega). cDNA was generated using oligo(dT)_20_ primer (GoScript Reverse Transcription System, Promega), before qPCR using primers for *socs3*, *c-fos* and *gapdh*, and iTaq Universal SYBR Green Supermix (Bio-Rad). Standard curves were generated for each primer pair using serial dilutions of the reference cDNA (samples from GFP-N-protein-expressing cells treated with OSM). Values were normalised to *gapdh* [6] and then calculated relative to that determined for untreated GFP-N-protein-expressing cells. Data from 3 independent assays were combined, where each assay result is the mean of replicate samples. Primers sequences were: 5’-GGTGCATTACAGAGAGGAGAAA-3’ and 5’ GTGTGTTTCACGCACAGATAAG-3’ for *c-fos;* 5’-GGAGTTCCTGGACCAGTACG-3’ and 5’-TTCTTGTGCTTGTGCCATGT-3’ for *socs3*; 5’-GAAGGTGAAGGTCGGAGTC-3’ and 5’-GGTCATGAGTCCTTCCACGAT-3’ for *gapdh*.

### Statistical analysis

Unpaired two-tailed Student’s *t*-test was performed using Prism software (version 7, GraphPad).

## Supporting information

S1 Data Excel spreadsheet containing, in separate sheets, the underlying numerical data for Fig panels 1B and S1, 2B, 2D, 3B, 3D, 4, 5B, 6, 7B, 8C, S2B, S5 and S6B. (XLSX)

S1 Fig Fn/c analysis of STAT3 in transfected cells lacking detectable expression of GFP. Images of COS7 cells used for the analysis shown in Fig 1 were analysed to determine the Fn/c for immunostained STAT3 in cells lacking detectable expression of the GFP-fused protein (GFP -) (mean ± SEM; n ≥ 33 cells for each condition). GFP + indicates analysis of cells with detectable GFP expression (data from Fig 1B, shown for comparison). Statistical analysis used Student’s *t* test. ****, p < 0.0001; NS, not significant.
(TIF)

S2 Fig RABV P-protein inhibits STAT3 responses to OSM, IL-6 and IFN-α. (A) COS7 cells transfected to express the indicated proteins were treated with or without OSM (10 ng/ml), IL-6 (100 ng/ml) or IFN-α (1000 U/ml) for 15 min before immunofluorescent staining for STAT3 (red) and analysis by CLSM as described in the legend to Fig 1. Representative images are shown. Arrowheads indicate cells with detectable expression of the transfected protein. Scale bars, 30 μm. (B) Images such as those shown in A were analysed to calculate the Fn/c for STAT3 (mean ± SEM, n ≥ 103 cells for each condition). Statistical analysis used Student’s *t* test. ****, p < 0.0001; NS, not significant.
(TIF)

S3 Fig STAT1 and STAT3 are activated to different extents by OSM and IFN-α. HEK293T cells were treated with or without OSM or IFN-α before lysis and IB analysis using antibodies against the indicated proteins, as described in the legend to Fig 3.
(TIF)

S4 Fig RABV P-protein interacts with STAT1 in OSM-treated 2fTGH cells. 2fTGH and U3A cells transfected to express GFP-CVS-Pwt were treated with or without OSM before immunoprecipitation of GFP and IB analysis using antibodies against the indicated proteins, as described in the legend to Fig 3. Results are from a single blot with intervening and marker lanes removed.
(TIF)

S5 Fig RABV P-protein antagonises OSM/STAT3-dependent gene expression. HEK293T cells co-transfected with m67-LUC and pRL-TK plasmids, and plasmids to express the indicated proteins, were treated with or without OSM before determination of relative luciferase activity (mean ± SEM; n = 4 independent assays, upper panel), as described in the legend to Fig 6; *lower panel*: cell lysates used in a representative assay were analysed by IB for GFP. Statistical analysis used Student’s *t* test. **, p < 0.01; ***, p < 0.001; ****, p < 0.0001.
(TIF)

S6 Fig Viral IFN-antagonists differentially regulate STAT3 responses to OSM. (A) COS7 and HeLa cells transfected to express the indicated proteins were treated with or without OSM before immunofluorescent staining for STAT3 (red) and analysis by CLSM as described in the legend to Fig 1. Representative images are shown. Arrowheads indicate cells with detectable expression of the transfected protein. Scale bars, 30 μm. (B) Images such as those shown in A were analysed to calculate the Fn/c for STAT3 (mean ± SEM, n ≥ 35 cells for each condition). Statistical analysis used Student’s *t* test. ****, p < 0.0001; NS, not significant.
(TIF)
