# Supplementary figures and images for: Lyssavirus P-protein selectively targets STAT3-STAT1 heterodimers to modulate cytokine signalling
Source: PLoS Pathog. 2020 Sep 9;16(9):e1008767. doi: 10.1371/journal.ppat.1008767 (PMC7480851; doi:10.1371/journal.ppat.1008767)

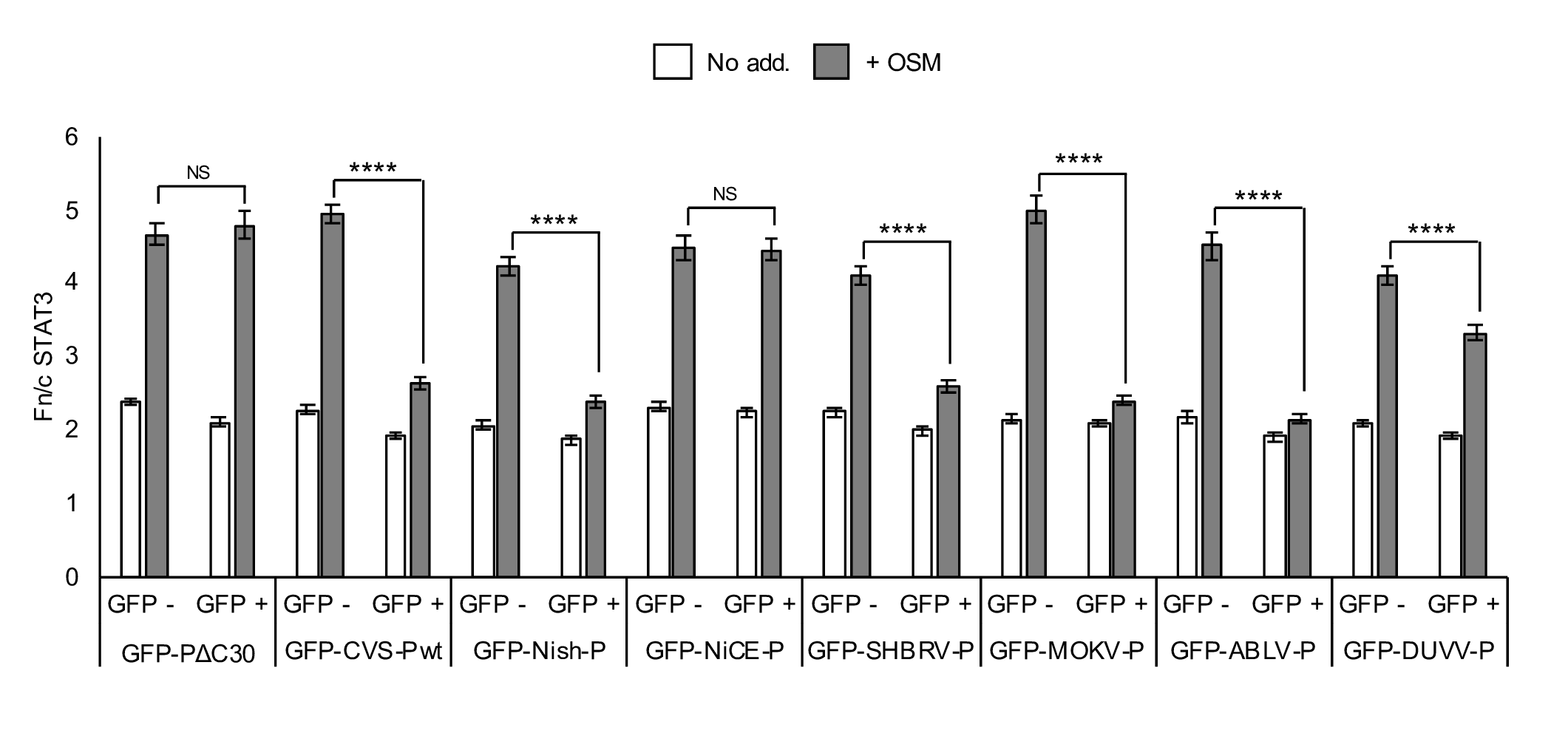

Supplement: S1 Fig — Images of COS7 cells used for the analysis shown in Fig 1 were analysed to determine the Fn/c for immunostained STAT3 in cells lacking detectable expression of the GFP-fused protein (GFP -) (mean ± SEM; n ≥ 33 cells for each condition). GFP + indicates analysis of cells with detectable GFP expression (data from Fig 1B, shown for comparison). Statistical analysis used Student’s t test. ****, p < 0.0001; NS, not significant. (TIF) [file ppat.1008767.s002.tif]

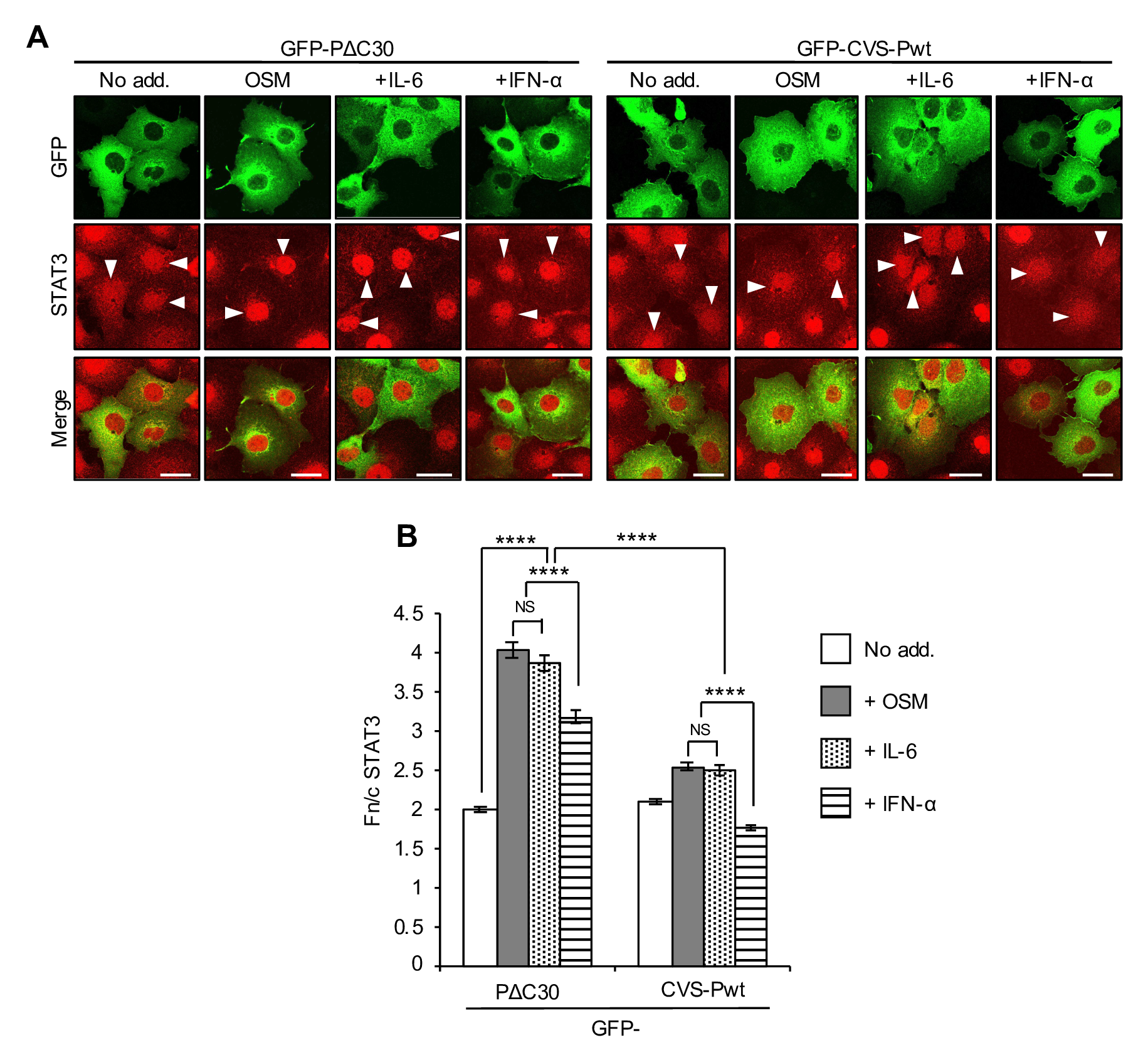

Supplement: S2 Fig — (A) COS7 cells transfected to express the indicated proteins were treated with or without OSM (10 ng/ml), IL-6 (100 ng/ml) or IFN-α (1000 U/ml) for 15 min before immunofluorescent staining for STAT3 (red) and analysis by CLSM as described in the legend to Fig 1. Representative images are shown. Arrowheads indicate cells with detectable expression of the transfected protein. Scale bars, 30 μm. (B) Images such as those shown in A were analysed to calculate the Fn/c for STAT3 (mean ± SEM, n ≥ 103 cells for each condition). Statistical analysis used Student’s t test. ****, p < 0.0001; NS, not significant. (TIF) [file ppat.1008767.s003.tif]

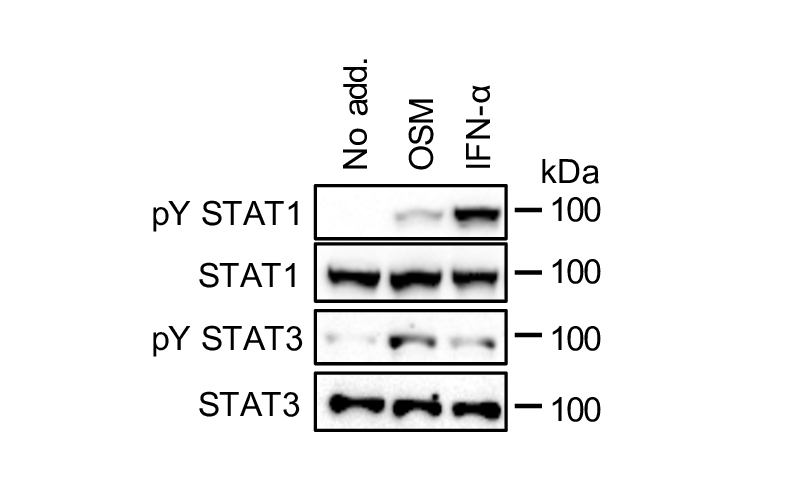

Supplement: S3 Fig — HEK293T cells were treated with or without OSM or IFN-α before lysis and IB analysis using antibodies against the indicated proteins, as described in the legend to Fig 3. (TIF) [file ppat.1008767.s004.tif]

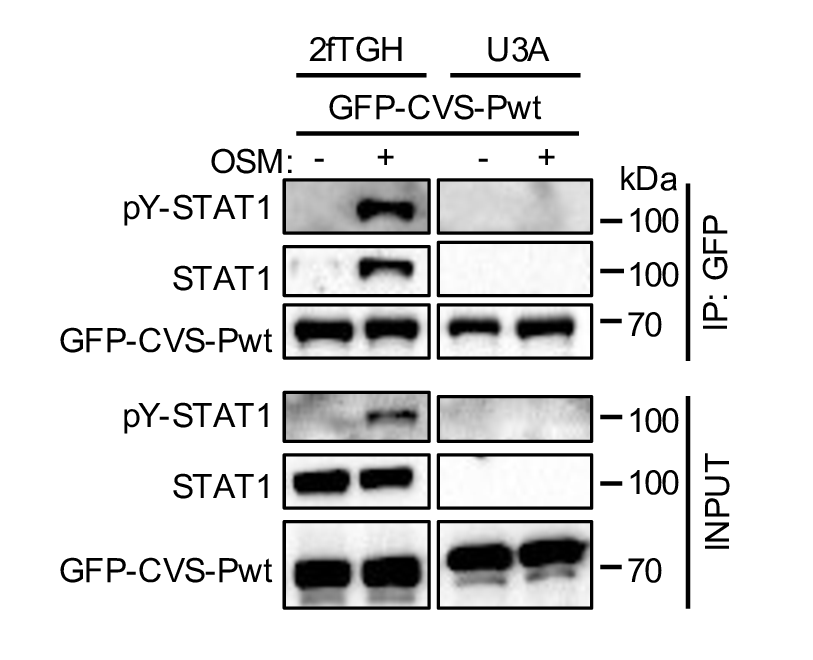

Supplement: S4 Fig — 2fTGH and U3A cells transfected to express GFP-CVS-Pwt were treated with or without OSM before immunoprecipitation of GFP and IB analysis using antibodies against the indicated proteins, as described in the legend to Fig 3. Results are from a single blot with intervening and marker lanes removed. (TIF) [file ppat.1008767.s005.tif]

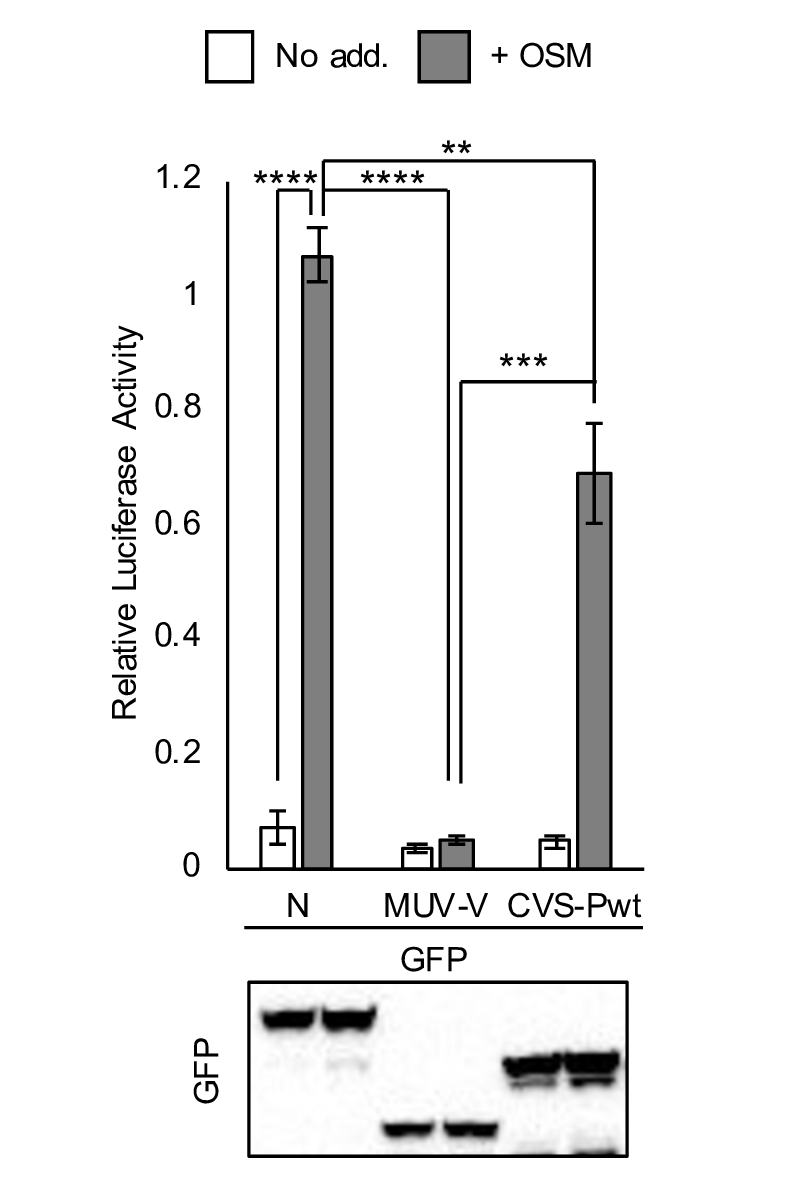

Supplement: S5 Fig — HEK293T cells co-transfected with m67-LUC and pRL-TK plasmids, and plasmids to express the indicated proteins, were treated with or without OSM before determination of relative luciferase activity (mean ± SEM; n = 4 independent assays, upper panel), as described in the legend to Fig 6; lower panel: cell lysates used in a representative assay were analysed by IB for GFP. Statistical analysis used Student’s t test. **, p < 0.01; ***, p < 0.001; ****, p < 0.0001. (TIF) [file ppat.1008767.s006.tif]

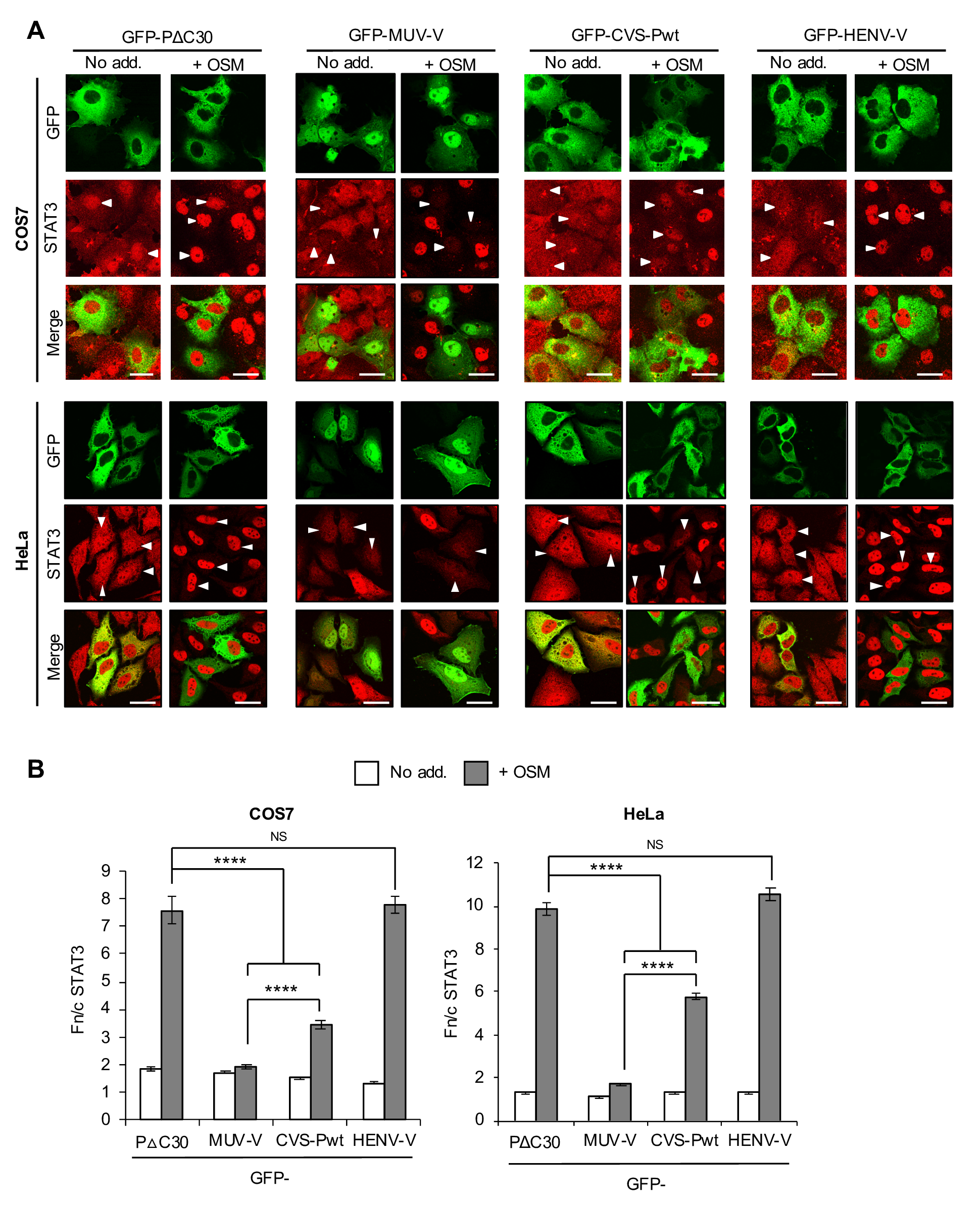

Supplement: S6 Fig — (A) COS7 and HeLa cells transfected to express the indicated proteins were treated with or without OSM before immunofluorescent staining for STAT3 (red) and analysis by CLSM as described in the legend to Fig 1. Representative images are shown. Arrowheads indicate cells with detectable expression of the transfected protein. Scale bars, 30 μm. (B) Images such as those shown in A were analysed to calculate the Fn/c for STAT3 (mean ± SEM, n ≥ 35 cells for each condition). Statistical analysis used Student’s t test. ****, p < 0.0001; NS, not significant. (TIF) [file ppat.1008767.s007.tif]
